# Long reaction times are associated with delayed brain activity in lewy body dementia

**DOI:** 10.1002/hbm.23866

**Published:** 2017-11-02

**Authors:** Michael J. Firbank, John T. O'Brien, John Paul Taylor

**Affiliations:** ^1^ Institute of Neuroscience, Newcastle University, Campus for Ageing and Vitality Newcastle upon Tyne NE4 5PL United Kingdom; ^2^ Department of Psychiatry University of Cambridge Cambridge United Kingdom

**Keywords:** bradyphrenia, Lewy body dementia, default mode network, reaction time

## Abstract

A significant symptom of Lewy body dementia (LBD) is slow cognitive processing or bradyphrenia. In a previous fMRI task‐based study, we found slower responses in LBD, accompanied by greater deactivation in the default mode network. In this study, we investigated the timing and magnitude of the activations and deactivations with respect to reaction time to determine whether the slower responses in LBD were associated with delayed neuronal activity. Using fMRI, we examined the magnitude and latency of activations and deactivations during an event‐related attention task in 32 patients with LBD and 23 healthy controls using predefined regions of interest. Default mode network deactivations did not significantly differ in their timing between groups or task conditions, while the task‐related activations in the parietal, occipital, frontal, and motor cortex were all significantly later in the LBD group. Repeating the analysis with reaction time as a parametric modulator of activation magnitude produced similar findings, with the reaction time modulator being significant in a number of regions including the default mode network, suggesting that the increased deactivation in LBD is partly explained by slower task completion. Our data suggest that the default mode network deactivation is initiated at the start of the task, and remains deactivated until its end, with the increased magnitude of deactivation in LBD reflecting the more prolonged cognitive processing in these patients. These data add substantially to our understanding of the neural origins of bradyphrenia, which will be essential for determining optimum therapeutic strategies for cognitive impairment in LBD. *Hum Brain Mapp 39:633–643, 2018*. © **2017 The Authors Human Brain Mapping Published by Wiley Periodicals, Inc.**

## INTRODUCTION

Lewy body dementia (LBD) includes both dementia with Lewy bodies and Parkinson's disease dementia, and is a major cause of dementia after Alzheimer's disease, representing 4–8% of all late onset dementia cases (Vann Jones and O'Brien, 2014). LBD is characterized by fluctuations in cognition, spontaneous motor features of parkinsonism, and complex visual hallucinations, and a wide array of other symptoms (Emre et al., 2007; McKeith et al., 2005). Deficits in attention and executive functioning are also a common feature in Lewy body dementia (Baddeley et al., 2001; Ballard et al., 2002; Ferman et al., 2006; Metzler‐Baddeley, 2007) and, in particular, cognitive slowing (or bradyphrenia) has been reported in both Parkinson's disease and Lewy body dementia, with slower response times in tasks, even after accounting for bradykinesia (motor slowing), as well as reduced information processing capability (Bailon et al., 2010; Bradshaw et al., 2006; Johnson et al., 2004; Sawamoto et al., 2002). Slow response times are associated with increased fluctuations in attention (Ballard et al., 2001), which has adverse effects on patient's and carer's quality of life (Bronnick et al., 2006; Lee et al., 2013). It has been suggested that bradyphrenia may be in part due to dopaminergic deficits in the basal ganglia (Jokinen et al., 2013), which play a role in determining the salience of, and reward associated with stimuli (Matsumoto, 2015). In addition the nicotinic receptors in the cholinergic system modulate information processing speed (Schneider et al., 2015; Wesnes and Warburton, 1984) and given the cholinergic system is profoundly affected in LBD (Bohnen et al., 2003; Tiraboschi et al., 2002) it is possible that dysfunction of this system may also play a role in slow cognitive speed in LBD. However, there is relatively little published research on the origins or neural correlates of cognitive slowing in LBD.

In a recent study (Firbank et al., 2016) using a version of the attention network task (ANT), we found slower reaction times in LBD compared to healthy controls, with increasing response latency for harder trials. In the LBD patients there was only limited evidence on fMRI analysis of increases in task‐related activations during more difficult aspects of the task. We hypothesized that the lack of increased BOLD activation suggested an absence of compensatory brain activity, and that the slower response was thus driven by delays in brain activation due to slower cognitive processing.

Subjects with LBD did, however, have significantly greater deactivation in the default mode network (DMN). This network, which includes the posterior cingulate, lateral parietal lobe and medial prefrontal cortex, is a network of regions which show high activity during rest, but decreased activity during a range of externally directed cognitive tasks. The DMN regions are thought to be involved in cognitive processes including episodic memory, internally directed attention and mind wandering (Andrews‐Hanna, 2012; Buckner et al., 2008). The deactivation of the DMN during tasks is believed to represent a decrease in internally directed thoughts (or mind wandering) as attention is paid to the task, and the DMN deactivation is greater during more difficult or more engaging tasks and thought to be necessary for optimising task performance (McKiernan et al., 2003; Arsalidou et al., 2013; Howard‐Jones et al., 2016). Studies of brain metabolism using blood flow SPECT and glucose metabolism PET have demonstrated profound reductions in the regions of the DMN including midline and lateral inferior parietal lobes of LBD patients (O'Brien et al., 2014). We hypothesised that in LBD, the increased deactivation of the DMN was due to the tasks being found more difficult by the subjects, owing to their cognitive and attentional deficits.

Our aim in this report was to investigate more thoroughly the association between response times in the LBD patients, and the relative timing of regional brain activity to see if delayed activation could play a role in the slow responses seen in the task. In particular, we wished to look at the latency and magnitude of the BOLD signal. Specifically, we hypothesized that

- the BOLD task positive network activations would be delayed in LBD relative to controls, increasingly so with harder condition, reflecting cognitive slowing;
- in both groups, the DMN deactivation would increase with harder condition, and that the deactivation would overall be greater in LBD, reflecting their finding the task more difficult;
- there would not be differences in DMN deactivation latency between conditions, as the deactivation occurs at the start of task, and thus is not dependent on difficulty.

## MATERIALS AND METHODS

### Participants

Subjects were the 32 Lewy body dementia (LBD) and 23 control subjects included in our previous study (Firbank et al., 2016). Participants were recruited prospectively from people aged over 60 with mild to moderate dementia with a Mini‐Mental State Examination (MMSE) score >12 from a local community‐dwelling population of participants who had been referred to local old age psychiatry and neurology services. Healthy controls were selected from friends and spouses of participants included in this and previous studies. The study was approved by the local ethics committee and written consent was obtained from all subjects.

Diagnosis of probable dementia with Lewy bodies, and Parkinson's disease with dementia was made independently by two experienced clinicians using the revised International Consensus Guidelines for dementia with Lewy bodies (McKeith et al., 2005) and diagnostic criteria for Parkinson's disease dementia (Emre et al., 2007), respectively. In accordance with our previous report, these two groups were combined into a LBD group. Cognitive function was tested using the Cambridge Cognitive Examination (CAMCOG, maximum score 105) and the MMSE (maximum score 30). Control participants in the study demonstrated no evidence of dementia (from history and score >80 on CAMCOG). Exclusion criteria for all participants included contra‐indications for MR imaging, moderate to severe visual impairment, previous history of alcohol or substance misuse, significant neurological or psychiatric history, moderate to severe cerebral small vessel disease, focal brain lesions on brain imaging, or the presence of other severe or unstable medical illness.

Before undergoing a scanning session and formal in‐scan testing with the ANT, participants were familiarized with the task, and it was verified that they could perform it correctly (task accuracy > 70%). All LBD patients were scanned whilst taking their usual anti‐parkinsonian medications and in an “ON” motor state, typically 1–3 h after last dose.

### Task

The task is described in detail in our previous report and is summarized in Supporting Information, Figure S1. Briefly, the task was based on the ANT (Fan et al., 2002) with a modified target component. On each trial, participants were shown a cue, followed by four arrowheads, and had to indicate the direction of the majority. The four arrowheads were either all pointing the same direction (congruent), or one of the arrows pointing the opposite direction (incongruent). The incongruent arrow appeared either on the end of the row (easy incongruent) or as one of the middle two (hard incongruent). Hence, the easy incongruent task had three congruent arrows in a row (unilateral flanker effect), whereas the hard task had only two (bilateral flanker effect). Behavioral contrasts were defined as (a) executive effect = mean RT of the incongruent (easy and hard) trials minus congruent trials and (b) conflict effect = mean RT of the hard incongruent minus easy incongruent trials.

### Neuroimaging Data Acquisition

Participants were scanned on a 3 T whole body MR scanner (Achieva scanner; Philips Medical System, the Netherlands), with body coil transmission and eight channel head coil receiver. Images acquired included a standard whole brain structural scan (3D MPRAGE, sagittal acquisition, slice thickness 1.0 mm, in plane resolution 1.0 × 1.0 mm; TR = 8.3 ms; TE= 4.6 ms; flip angle = 8°; SENSE factor = 2). fMRI data were collected with a gradient‐echo (GE) echo planar imaging (EPI) sequence (TR = 1.92 s; TE = 40 ms; field of view (FOV) = 192 × 192 mm^2^ 64 × 64 matrix size, flip angle 90°, 27 slices, slice thickness 3 mm, slice gap 1 mm) with 156 volumes (5 min). We collected between four and six runs of fMRI data while participants performed the attention task. We excluded those runs with <2/3 correct responses, as performance per run worse than this was not significantly different from chance.

### fMRI Analysis and Statistics

We used SPM8 (http://www.fil.ion.ucl.ac.uk/spm/) for all image analysis. As described previously (Firbank et al., 2016), the fMRI data were motion corrected and normalized to MNI space via alignment with the T1 scan and the DARTEL toolbox (Ashburner, 2007). A high pass filter of 128 s was used, and serial correlations were removed with SPM's AR(1) model.

Runs with >3 mm or >3° head motion were excluded. We did not perform any interpolation or “scrubbing” of bad image volumes. To investigate possible influences of data quality, we calculated the mean and maximum absolute angular and translational motion between frames (Peraza et al., 2015). We also calculated standardized DVARS—the per‐image standard deviation of the temporal derivative of the data (Power et al., 2012) using Tom Nichols's script (http://www2.warwick.ac.uk/fac/sci/statistics/staff/academic-research/nichols/scripts/fsl/DVARS.sh) and determined the mean and maximum over all volumes in each run.

The general linear model (GLM) in SPM was used to conduct a whole‐brain analysis of the fMRI data. We created a design matrix using an impulse function with onset time of the events (cues and targets with correct responses). Missed targets and incorrectly responded to targets were combined as an extra column in the design matrix. These events were convolved with the canonical hemodynamic response function (HRF), and the first derivative of the HRF was also included to model variation in onset latency. The six parameters from the motion correction for each functional run were included in the design matrix as covariates of no interest. The regressors were fitted to the fMRI data to produce beta estimates for each regressor.

To further investigate the variation in BOLD with RT within conditions, we performed a GLM with the addition of parametric modulation; for each of the 3 task conditions, an additional variable was included consisting of the HRF onset intensity for each trial modulated by the RT for that trial.

We used the formula of Henson et al (Henson et al., 2002) to determine BOLD latency from the first derivative of the HRF.

BOLD latency = 2×1.78/(1+exp(3.1×beta_2/beta_1))−1.78

where beta_1 and beta_2 are the estimates of the HRF and its first derivative, respectively. This was calculated on a voxelwise basis from the SPM beta images using an in‐house Matlab script (available on https://www.nitrc.org/projects/sigmoidal_dt/).

We performed all analysis using regions of interest (ROI) as defined previously (Firbank et al., 2016), taken from the incongruent vs congruent contrast derived from all participants (dementia patients and healthy controls). The activation regions comprised a number of distinct clusters, with some connection between clusters. They were manually divided into distinct anatomical regions based on the clusters (mid frontal, lateral frontal, insula, parietal, occipital), and the deactivations into frontal (medial anterior) and posterior (mostly posterior cingulate). These ROIs are shown in Supporting Information, Figure S2 and Table S1. In addition, we defined a motor area region from the combination of pre and post central gyrus regions from the AAL atlas (Tzourio‐Mazoyer et al., 2002). We utilized the MarsBaR SPM toolbox (http://marsbar.sourceforge.net/) to extract mean values for the BOLD contrast for the comparisons. The fMRI ROI data were analyzed with a repeated measures ANOVA, with each region investigated separately. Greenhouse–Geisser correction for nonsphericity was used for within subject tests.

The SPSS (version 23, IBM) package was used to calculate all statistics. In order to control for multiple comparisons in the ROI analysis, we used a Bonferroni correction (factor of 9, giving an adjusted *P* < 0.0056).

## RESULTS

Table 1 shows the subject demographics. There were no significant differences in age, sex, or years of education between the LBD and control participants. As previously reported, (Firbank et al., 2016) RT were overall longer in LBD (congruent RT = 1125 ms, SD 262 ms; easy incongruent RT 1521 ms, SD 391 ms; hard incongruent RT 1800 ms, SD 486 ms) vs control (congruent RT = 714 ms, SD 121 ms; easy incongruent RT = 1005 ms, SD 217 ms; hard incongruent RT = 1084 ms, SD 265 ms), with an increased executive/conflict RT effect of 200 ms. After exclusion of fMRI runs with excess motion or poor responses, 6 runs were included for 21/23 controls and 24/32 LBD; 5 runs for 2 control and 5 LBD; and 4 runs for 3 LBD (no significant group difference in number of runs *χ* ^2^ =2.13, *P* = 0.12). There were no group differences in mean or maximum translational movement (Table 1). The mean angular motion was slightly greater in the DLB group, but there were no differences in the maximum angular movement. The maximum and mean of the DVARS measure did not differ between groups.

**Table 1:** Demographics and clinical scores

	Controls (*N* = 23)	LBD (*N* = 32)	Between‐group differences (*P* value)
Age	76.3 (5.5)	75.0 (6.4)	*T*_53_ = 0.76; *P* = 0.45
Sex male:female	16:7	27:5	*χ* ^2^ = 1.7; *P* = 0.2
Education years	11.7 (2.3)	10.5 (2.5)	*T*_53_ = 1.9; *P* = 0.06
Duration (years) cognitive decline	‐	3.33 (2.07)	
Cholinesterase Inhibitors	0 (0%)	27 (84%)	‐
Ldopa equivalent dose	‐	683.9 (450.1) [*N* = 21]	
Positive DAT scan	‐	10/11 (91%)	
UPDRS	1.3 (1.7)	19.06 (8.0)	
MMSE	29.1 (0.9)	23.4 (3.8)	
CAMCOG	96.8 (3.6)	76.7 (12.6)	
Mean xyz motion (mm)	0.139 (0.04)	0.145 (0.08)	*T*_53_ = 0.33; *P* = 0.74
Max xyz motion (mm)	0.602 (0.27)	0.618 (0.31)	*T*_53_ = 0.21; *P* = 0.84
Mean angular motion (°)	0.062 (0.012)	0.088 (0.060)	*T*_53_ = 2.07; *P* = 0.043
Max angular motion (°)	0.463 (0.28)	0.491 (0.28)	*T*_53_ = 0.36; *P* = 0.72
Mean DVARS	1.36 (0.14)	1.36 (0.19)	*T*_53_ = 0.01; *P* = 1.0
Max DVARS	4.32 (1.76)	3.85 (1.18)	*T*_53_ = 1.18; *P* = 0.24

CAMCOG, Cambridge Cognitive Examination; MMSE, Mini‐Mental State Examination; UPDRS, Unified Parkinson's disease rating scale.

DVARS per‐image standard deviation of the temporal derivative.

**Figure 1:**
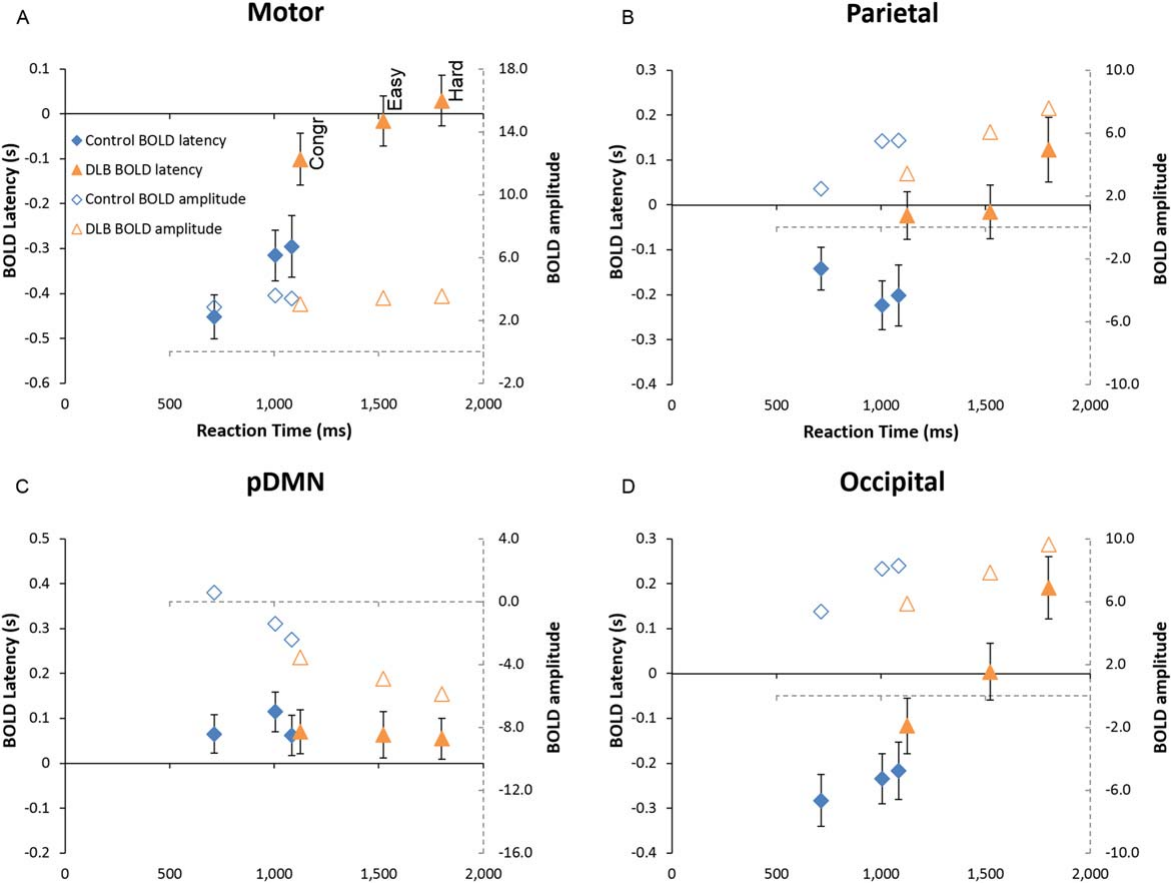
Plot of BOLD latency and activation magnitude against reaction time to illustrate the repeated measures analysis presented in Tables 2 and 3, for the ROIs in the (A) motor cortex, (B) parietal lobe, (C) posterior default mode network (pDMN), and (D) occipital lobe. The 3 points per group (from left to right) are the congruent, easy, and hard incongruent conditions. Solid symbols are BOLD latency in seconds (on the left‐hand axis) and empty symbols are BOLD magnitude (on the right‐hand axis, with the dashed lines). The total range of the *x*‐axes is the same for all subplots. [Color figure can be viewed at http://wileyonlinelibrary.com]

The results from the BOLD analysis are shown in Tables 2 and 3 and Figure 1. For the BOLD amplitude, the repeated measures ANOVA showed an overall effect of condition (as expected from the ROI definition), with an effect of group only on the DMN regions (significant in pDMN after Bonferroni correction), consistent with the increased deactivation of the DMN in the LBD group. For the latency (Table 3), however, there was a significant effect of group (with LBD having a longer BOLD latency) for all but the DMN and midfrontal regions. The motor area showed an effect of condition on latency, with BOLD latency increasing from congruent to easy‐incongruent to hard‐incongruent paralleling the increase in RT in these conditions. Occipital regions had a similar effect of condition on latency, as well as demonstrating a significant interaction between group and condition, with the incongruent trials having relatively increased latency for the LBD group. There were also significant interactions (*P* ∼= 0.04, not surviving Bonferroni correction) between condition and group for the BG region for both latency and amplitude, the midfrontal for BOLD amplitude, and the insula for BOLD latency. These interactions were all of greater increase in BOLD amplitude and latency with the harder conditions for LBD.

**Table 2:** BOLD Magnitude for the Three Conditions (vs Rest). Values are Mean (SD)

	Control			LBD			Repeated measures		
	Congruent	Incongr easy	Incongr hard	Congruent	Incongr easy	Incongr hard	Group	Condition	Condition × Group
Mid frontal	1.85 (2.81)	4.07 (3.18)	3.80 (3.61)	2.77 (2.90)	4.87 (4.53)	6.13 (4.09)	*F*_1,53_ = 2.16; *P* = 0.147	*F*_2.0,105.2_ = 38.01; *P* = 0.000^**^	*F*_2.0,105.2_ = 3.46; *P* = 0.036^*^
Lateral frontal	0.97 (2.34)	3.41 (3.01)	3.40 (3.10)	1.42 (2.52)	3.35 (3.08)	4.72 (3.22)	*F*_1,53_ = 0.61; *P* = 0.437	*F*_1.8,94.9_ = 57.65; *P* = 0.000^**^	*F*_1.8,94.9_ = 3.11; *P* = 0.055
Insula	2.47 (3.15)	5.76 (3.15)	5.45 (3.59)	2.25 (3.30)	3.93 (3.96)	5.24 (4.67)	*F*_1,53_ = 0.65; *P* = 0.422	*F*_1.8,97.6_ = 36.91; *P* = 0.000^**^	*F*_1.8,97.6_ = 3.13; *P* = 0.052
Parietal	2.47 (2.13)	5.48 (2.91)	5.54 (3.28)	3.43 (3.39)	6.07 (4.42)	7.60 (4.58)	*F*_1,53_ = 1.66; *P* = 0.203	*F*_1.7,92.4_ = 72.52; *P* = 0.000^**^	*F*_1.7,92.4_ = 2.92; *P* = 0.066
Occipital	5.36 (3.57)	8.11 (3.71)	8.28 (4.50)	5.88 (2.95)	7.87 (4.35)	9.65 (4.75)	*F*_1,53_ = 0.29; *P* = 0.595	*F*_1.8,93.9_ = 50.36; *P* = 0.000^**^	*F*_1.8,93.9_ = 2.75; *P* = 0.075
pDMN	0.57 (3.30)	−1.39 (3.58)	−2.40 (4.20)	−3.53 (3.02)	−4.90 (3.46)	−5.87 (4.14)	*F*_1,53_ = 16.53; *P* = 0.000^**^	*F*_2.0,104.1_ = 30.04; *P* = 0.000^**^	*F*_2.0,104.1_ = 0.52; *P* = 0.591
fDMN	−0.08 (2.22)	−1.08 (2.35)	−1.68 (2.70)	−2.68 (3.49)	−3.82 (4.56)	−4.08 (5.17)	*F*_1,53_ = 7.10; *P* = 0.010^*^	*F*_2.0,105.1_ = 13.45; *P* = 0.000^**^	*F*_2.0,105.1_ = 0.17; *P* = 0.843
Motor Cortex	2.87 (1.26)	3.60 (1.59)	3.41 (1.81)	3.03 (1.57)	3.45 (1.69)	3.53 (1.65)	*F*_1,53_ = 0.01; *P* = 0.918	*F*_1.9,98.5_ = 12.43; *P* = 0.000^**^	*F*_1.9,98.5_ = 0.91; *P* = 0.401
BG	3.44 (2.89)	5.47 (3.11)	4.91 (3.50)	2.46 (2.86)	4.11 (4.20)	5.13 (3.27)	*F*_1,53_ = 0.69; *P* = 0.409	*F*_2.0,104.0_ = 26.83; *P* = 0.000^**^	*F*_2.0,104.0_ = 3.52; *P* = 0.034^*^

**P < 0.0056 (Bonferroni correction); *P < 0.05.

DMN, default mode network; BG, basal ganglia.

**Table 3:** BOLD Latency in Seconds for the Three Conditions. Values are Mean (SD)

	Control			LBD			Repeated measures		
	Congruent	Incongr easy	Incongr hard	Congruent	Incongr easy	Incongr hard	Group	Condition	Condition × Group
Mid frontal	−0.125 (0.258)	−0.249 (0.274)	−0.214 (0.288)	−0.119 (0.255)	−0.140 (0.253)	−0.118 (0.270)	*F*_1,53_ = 1.39; *P* = 0.243	*F*_1.7,89.7_ = 2.06; *P* = 0.141	*F*_1.7,89.7_ = 1.23; *P* = 0.294
Lateral frontal	−0.094 (0.230)	−0.151 (0.182)	−0.138 (0.183)	0.024 (0.203)	−0.021 (0.214)	0.014 (0.209)	*F*_1,53_ = 9.51; *P* = 0.003^**^	*F*_1.6,84.6_ = 1.37; *P* = 0.257	*F*_1.6,84.6_ = 0.15; *P* = 0.817
Insula	−0.030 (0.220)	−0.099 (0.199)	−0.073 (0.225)	0.011 (0.207)	0.060 (0.171)	0.134 (0.206)	*F*_1,53_ = 10.46; *P* = 0.002^**^	*F*_2.0,104.9_ = 1.39; *P* = 0.255	*F*_2.0,104.9_ = 3.64; *P* = 0.030^*^
Parietal	−0.142 (0.228)	−0.223 (0.260)	−0.202 (0.328)	−0.023 (0.299)	−0.015 (0.338)	0.123 (0.407)	*F*_1,53_ = 9.03; *P* = 0.004^**^	*F*_1.5,79.5_ = 1.71; *P* = 0.194	*F*_1.5,79.5_ = 2.84; *P* = 0.079
Occipital	−0.283 (0.277)	−0.234 (0.268)	−0.217 (0.307)	−0.117 (0.349)	0.004 (0.357)	0.191 (0.390)	*F*_1,53_ = 10.71; *P* = 0.002^**^	*F*_1.8,93.3_ = 15.24; *P* = 0.000^**^	*F*_1.8,93.3_ = 6.75; *P* = 0.003^**^
pDMN	0.065 (0.205)	0.115 (0.213)	0.062 (0.216)	0.070 (0.277)	0.064 (0.291)	0.055 (0.256)	*F*_1,53_ = 0.10; *P* = 0.750	*F*_1.9,100.9_ = 0.39; *P* = 0.670	*F*_1.9,100.9_ = 0.33; *P* = 0.706
fDMN	0.041 (0.184)	0.091 (0.154)	0.083 (0.199)	−0.025 (0.252)	0.012 (0.259)	0.031 (0.236)	*F*_1,53_ = 1.98; *P* = 0.165	*F*_1.9,99.4_ = 1.23; *P* = 0.295	*F*_1.9,99.4_ = 0.08; *P* = 0.914
Motor Cortex	−0.452 (0.231)	−0.315 (0.270)	−0.295 (0.330)	−0.101 (0.327)	−0.016 (0.317)	0.030 (0.319)	*F*_1,53_ = 18.52; *P* = 0.000^**^	*F*_1.7,87.6_ = 12.27; *P* = 0.000^**^	*F*_1.7,87.6_ = 0.36; *P* = 0.655
BG	−0.133 (0.209)	−0.178 (0.229)	−0.149 (0.191)	−0.020 (0.178)	0.077 (0.178)	0.040 (0.222)	*F*_1,53_ = 17.58; *P* = 0.000^**^	*F*_2.0,103.5_ = 0.49; *P* = 0.607	*F*_2.0,103.5_ = 3.18; *P* = 0.047^*^

**P < 0.0056 (Bonferroni correction); *P < 0.05.

DMN, default mode network; BG, basal ganglia.

Supporting Information, Tables 2–4 shows the repeated measures ANOVA results from the SPM analysis including the RT parametric modulation. The results for the amplitude and latency were similar to the original analysis, with both DMN ROIs showing an effect of group on BOLD amplitude, but no difference in latency, whilst latency differences are still present for the insula, parietal and occipital regions. For the RT parametric modulator of BOLD magnitude, there were no significant differences between groups or conditions, but there was an overall significant effect, with longer RT within each condition being associated with increased BOLD in the task positive ROIs, and with increased deactivation in the pDMN (and trend in the fDMN).

As a secondary analysis we also investigated the potential effect of levodopa, by looking at Spearman correlations between the levodopa equivalent dose and the BOLD activity and latency in all regions and conditions. Significant correlations (not surviving Bonferroni correction) were seen only in latency during easy incongruent for midfrontal (*ρ* = −0.36, *P* = 0.043) and insula (*ρ* = −0.35, *P* = 0.048) ROI. There were no significant correlations in the LBD group between UPDRS motor score and reaction time, BOLD amplitude or latency. There were also no significant differences between those LBD patients taking cholinesterase inhibitors vs not in response time or BOLD amplitude or latency.

To investigate the potential effect of group differences in data quality, we repeated the analysis in Tables 2 and 3 using only those participants with all 6 runs, and with mean angular and translational motion within 3SD of the control group (21/23 controls, and 18/32 LBD). This produced qualitatively similar results, with the only difference being that the fDMN had an effect of condition on BOLD latency (*F*_2,74_ = 4.1; *P* = 0.021) although this was not significant after Bonferroni correction.

## DISCUSSION

Overall, our findings suggest that in this attention task, the LBD group have slower cognitive processing, with task related BOLD activations in the parietal, occipital, frontal, insula and motor cortex starting later in LBD compared to controls. In contrast, the deactivations in the DMN regions seemed to start at the same time regardless of task or group, and were maintained until a response was made.

As we previously reported (Firbank et al., 2016), analysis of the reaction time data showed that the LBD group were both slower overall, (congruent RT *T*_53_ = 7.0; *P* < 0.001) and disproportionately slower for harder tasks (incongruent–congruent RT *T*_53_ = 3.9, *P* < 0.001) compared to controls. There was no significant correlation between UPDRS motor score and reaction time, suggesting lack of a direct influence of motor problems on the responses. The analysis of the ROI data from the SPM analysis (Tables 2 and 3) showed that the overall amplitude of the task positive BOLD activations increased with difficulty, but not across group. However, the latency of the BOLD response was greater in the LBD for most of the ROIs. In the second SPM analysis, controlling for the effect of RT within condition (Supporting Information, Tables 2–4), the same general pattern was seen, and in addition, a number of regions (insula, parietal, BG, and occipital) also showed an interaction between condition and group, such that the harder conditions had relatively increased latency for the LBD compared to controls. These results suggest that the increased RT in the LBD group is a result of delayed onset of task related cognitive activity, and hence slower processing. These regions are all affected in LBD, with metabolic reductions in the occipital and parietal lobes (Firbank et al., 2017; Kantarci et al., 2012), reports of reduced grey matter in the insula (Blanc et al., 2016; Xu et al., 2016) and cholinergic (Mazère et al., 2017) and dopaminergic (O'Brien et al., 2009) alterations in the BG.

For the default mode network regions, however, a different effect was seen. The BOLD onset latency did not differ either with task difficulty, or between LBD and controls. The magnitude of the deactivation was significantly different between groups and condition, but in the parametric modulator analysis, (Supporting Information, Table S4) which found a significant modulatory effect of the RT on the magnitude of the pDMN deactivation (increased RT associated with greater deactivation), there was no difference in the modulation between groups or conditions, implying that the increased BOLD deactivation in LBD and harder conditions was simply an effect of more prolonged cognitive processing. Altogether, this suggests that the BOLD deactivation is initiated at the start of each trial, and lasts until the end of the task related cognitive processing.

The relationship between DMN deactivation and task performance is dependent upon the nature of the task. Increases in DMN activity are present during self‐reflection; in memory tasks, deactivation of the DMN has been associated with better encoding (where deactivation represents suppression of internal thoughts, and greater attention on the task), but worse recall (as activation of the DMN is required for recall) (Daselaar et al., 2009). In general, for externally oriented tasks, more difficult, attention demanding tasks have greater deactivation of the DMN (Andrews‐Hanna, 2012) which is in keeping with our observations. So for an individual, for tasks of a fixed difficulty, increased deactivation is likely to be associated with shorter RT, while increasing difficulty will be associated with increased deactivation and longer RT. Unlike AD, where there is dysfunctional behavior of the DMN (Anticevic et al., 2012; Firbank et al., 2016), our findings here suggest that in LBD, the DMN is fundamentally intact, showing deactivations during externally directed behavior, with the magnitude of the deactivation in keeping with the duration of the trials. The primary difference in the DMN in LBD is that the deactivations are more profound, which we ascribe to slower cognitive functioning elsewhere in the brain. Our findings are in agreement with a previous study, which found normal DMN activity, but altered functional connectivity in the frontal and parietal lobes of DLB (Franciotti et al., 2013). Similarly, in an analysis on the group presented here (Kobeleva et al., 2017), we found reduced connectivity between the ventral attention network and dorsal attention network in LBD, with normal connectivity of the DMN.

Slower cognition or bradyphrenia is a significant feature of Lewy body diseases, and has been reported in dementia free Parkinson's disease subjects, controlling for bradykinesia (Sawamoto et al., 2002). Visual inspection time, which does not have a motor component, has been found to be significantly increased in Parkinson's disease (Johnson et al., 2004), and DLB (Bailon et al., 2010), suggesting that visual processing is slower in Lewy body disease. Using neuroimaging, slower cognitive processing in Parkinson's disease has been associated with abnormal caudate perfusion, (Sawamoto et al., 2007) and dopamine changes in the caudate and anterior cingulate have been linked with reaction time (Jokinen et al., 2013) and working memory deficits in Parkinson's disease (Sawamoto et al., 2008). In keeping with these neuroimaging reports in Parkinson's disease, we observed an interaction between BOLD magnitude and group (Table 2) in the mid frontal (including anterior cingulate) and basal ganglia regions such that the LBD had a disproportionate increase in BOLD for harder conditions. The caudate is involved in motor and cognitive initiation (Grahn et al., 2008) and thus our findings are consistent with the argument that disruption of the dopaminergic system via the basal ganglia may be, in part, responsible for the slow processing in LBD. However, the cholinergic system, which influences attention and information processing speed (Schneider et al., 2015; Wesnes and Warburton, 1984) is also dysfunctional in LBD, and the cholinergic projections from the brainstem to the thalamus and basal ganglia have been found to be particularly affected in LBD compared to AD (Kotagal et al., 2012).

Dopaminergic treatment can alter brain network function, and levodopa has been reported to enhance functional connectivity (Krajcovicova et al., 2012) of the posterior DMN. Our patients were all scanned whilst taking their dopaminergic anti‐parkinsonism medication (although not all participants were on these agents), which may have affected responses and DMN deactivation. However, recent studies in cognitively intact Parkinson's disease have found no effect of levodopa on choice reaction times, despite improvements to bradykinesia (Michely et al., 2012; Michely et al., 2015). Similarly, the reported effect of levodopa is to restore to normality a reduced DMN deactivation, rather than to enhance it (Delaveau et al., 2010; Spetsieris et al., 2015). Thus we believe that the slow RT and greater DMN deactivation we see are unlikely to be due to dopaminergic medication. Cholinergic drugs have been reported to improve attentional function (Bentley et al., 2003; Broks et al., 1988; McKeith et al., 2000), largely by modulating frontoparietal networks (Bentley et al., 2011; Bokde et al., 2009; Risacher et al., 2013). Most of the LBD patients were taking cholinesterase inhibitor medication, which has been shown to result in faster RT in Parkinson's disease (Rowan et al., 2007), so may have reduced response latencies in subjects, although we did not see any differences in those patients taking such medication compared to those not on these agents. Further studies are needed to investigate the role of cholinergic and dopaminergic medication in cognitive tasks in LBD to help clarify the neuronal origin of cognitive slowing.

Examining the occipital lobe BOLD latency, we observed a significant interaction between condition and group (Table 3), with the LBD patients having increased latency for the harder conditions. One would expect the onset of BOLD activation of the occipital lobe to occur in response to stimulus presentation, as the visual system processes the incoming event (Corbetta and Shulman, 2002). We postulate that the increased latency in the occipital lobe with harder conditions is due to top–down influences, (Siemann et al., 2016) as the target is probed more carefully in the conflict conditions, leading to an ongoing rise in occipital activity. This increase was present in both groups, but was more marked in the LBD patients, perhaps reflecting inefficient visual processing, and/or top–down visuo‐attentional deficits. If so, this inefficient processing would also contribute to the slower response time in the LBD subjects. Further analysis using techniques like dynamic causal modeling (Friston et al., 2003) is required to investigate how the relative timing of different regional activity contributes to cognitive slowing in LBD.

In the motor cortex, as expected, the latency of the BOLD response varied with condition (with the latency increasing for the conditions with longer RT) and group (with the LBD group having later latency than controls). This is in keeping with later initiation of motor activity for those more difficult trials with resultant longer reaction times. The delayed start of motor activity in LBD also adds support to the idea that the longer reaction times in LBD are partly due to slower cognition rather than motor impairments/bradykinesia.

Limitations include that we did not correct for slice timing effects in our fMRI processing. However, the slice prescription was the same for all subjects, so the relative position of each slice in the brain should be approximately the same for all. In particular, the posterior DMN region (which showed no change in BOLD latency) is immediately adjacent to the parietal region (which did show a significant difference). In addition, the BOLD signal is an indirect measure of neuronal activity, and delays in the timing of the BOLD may reflect, for instance, differences in cardiovascular responses between LBD patients and our comparison subjects. Many LBD subjects were taking cholinergic and/or dopaminergic medication, which may have altered brain connectivity. The posterior DMN regions show a decrease in resting glucose metabolism and blood flow in PD, which is more marked in subjects with cognitive decline (Bohnen et al., 2011; Firbank et al., 2017; Huang et al., 2007). Owing to the highly connected nature of the region, it remains unclear whether cognitive related changes reflect intrinsic pathology, or alterations in response to change elsewhere. Although our results found that the latency of the BOLD activation was increased in LBD, we cannot unambiguously ascribe the RT delays to this presumed neuronal delay. In particular, we found differences in latency in the basal ganglia, which are involved in both motor and cognitive functions. Further studies investigating functional connectivity are required to determine how the basal ganglia interacts with other regions in this task, to clarify to what extent the slow RT is due to BG input to motor regions, and how much to its involvement in cognitive slowing.

Overall, our finding that brain activation in LBD was relatively delayed across regions involved in the task suggests that the slow reaction times in LBD in the attention network task are a result of slower cognitive processes rather than purely bradykinesia. In comparison, the task‐related deactivation of the default mode network in LBD seems essentially intact, but the longer decision time leads to a greater deactivation. Our data showing an interaction of basal ganglia activity with group/task are in keeping with a possible role for dysfunction of the dopaminergic and/or cholinergic system in slower cognition.

## Supporting information

Supporting Information Figure 1

Supporting Information Figure 2

Supporting Information
