# Supplementary figures and images for: Long reaction times are associated with delayed brain activity in lewy body dementia
Source: Hum Brain Mapp. 2017 Nov 2;39(2):633–43. doi: 10.1002/hbm.23866 (PMC5813138; doi:10.1002/hbm.23866)

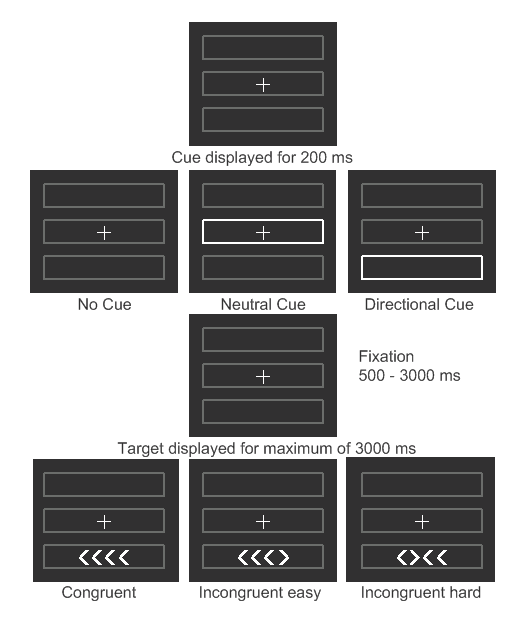

Supplement: Supplementary file 1 — Supporting Information Figure 1 [file HBM-39-633-s001.tif]

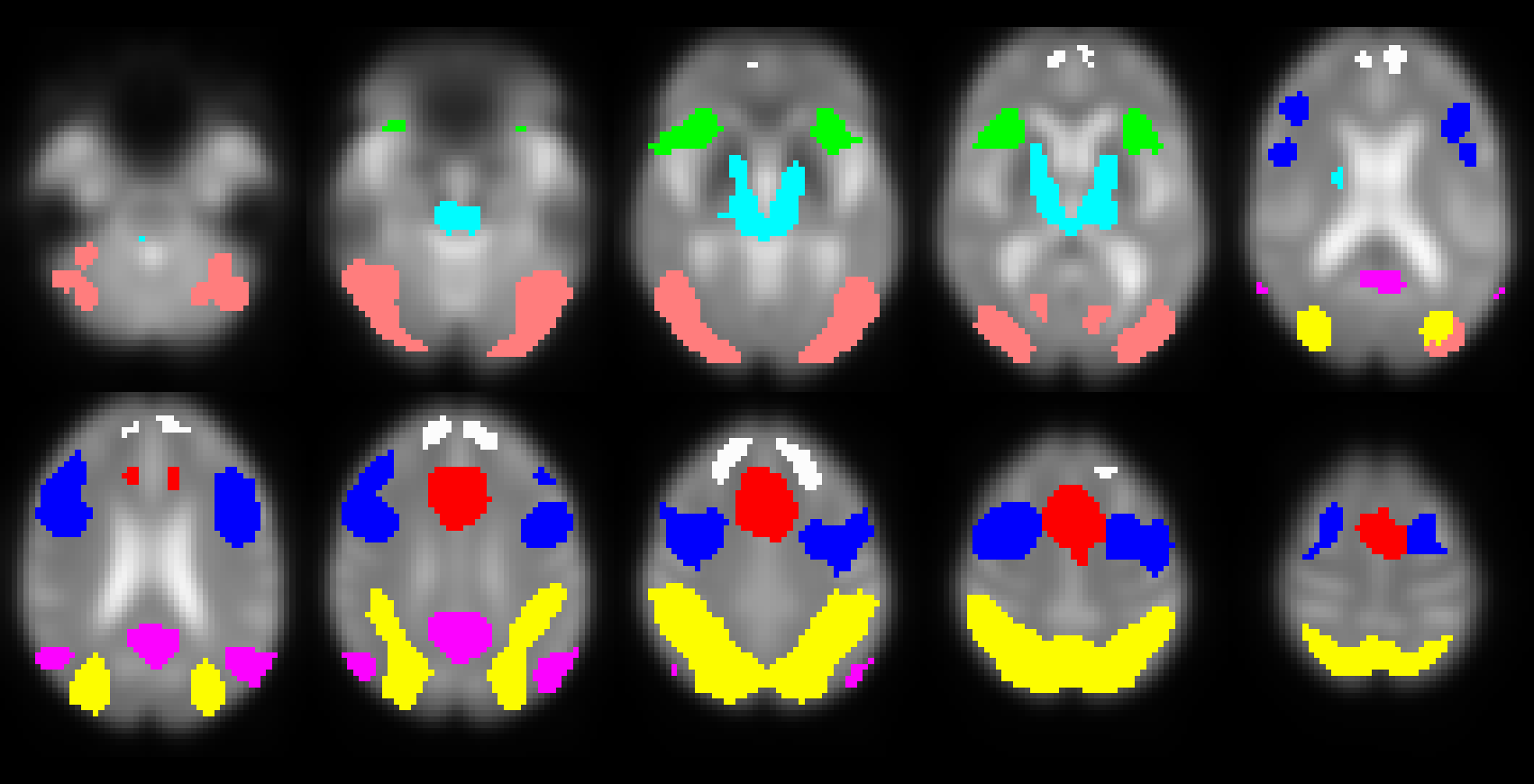

Supplement: Supplementary file 2 — Supporting Information Figure 2 [file HBM-39-633-s002.tif]
