# Supplementary material for: Long reaction times are associated with delayed brain activity in lewy body dementia
Source: Hum Brain Mapp. 2017 Nov 2;39(2):633–43. doi: 10.1002/hbm.23866 (PMC5813138; doi:10.1002/hbm.23866)
Supplement: Supplementary file 3 — Supporting Information [file HBM-39-633-s003.doc]

**SUPPLEMENTARY TABLES**

Table S1 Location and volume of ROIs

|  | Voxels | COG X (mm) | COG Y (mm) | COG Z (mm) |
| --- | --- | --- | --- | --- |
| Mid frontal | 1244 | 0.427 | 15.3 | 45.9 |
|  |  |  |  |  |
| Lateral frontal L | 1256 | -37.7 | 7.16 | 39.3 |
| Lateral frontal R | 1374 | 41.1 | 12.3 | 38.1 |
|  |  |  |  |  |
| Insula L | 262 | -33.8 | 21.8 | -1.19 |
| Insula R | 346 | 38.5 | 21.9 | -2.19 |
|  |  |  |  |  |
| Parietal L | 1876 | -27.3 | -59.6 | 43.2 |
| Parietal R | 2068 | 29.9 | -58.8 | 44.1 |
|  |  |  |  |  |
| Occipital L | 1218 | -38 | -73.2 | -9.65 |
| Occipital L V1 | 50 | -11.2 | -74.2 | 5.34 |
| Occipital R | 1111 | 38.9 | -69.9 | -9.61 |
| Occipital R V1 | 50 | 15 | -69.4 | 6.6 |
|  |  |  |  |  |
| DMN anterior L | 309 | -12.6 | 49.9 | 32.5 |
| DMN anterior R | 225 | 14.6 | 50.7 | 31.6 |
|  |  |  |  |  |
| DMN parietal L | 293 | -49.1 | -66.5 | 30.2 |
| DMN parietal R | 152 | 51.6 | -63.4 | 29.3 |
| DMN post cingulate | 510 | -0.712 | -51.5 | 27.3 |

Table S2 BOLD magnitude for the 3 conditions or the SPM model with RT included as a parametric modulator.

|  | Control |  |  | LBD |  |  |  |  |  |
| --- | --- | --- | --- | --- | --- | --- | --- | --- | --- |
|  | N=23 |  |  | N=32 |  |  |  |  |  |
|  |  |  |  |  |  |  |  |  |  |
|  | Congruent | Incongruent easy | Incongruent  hard | Congruent | Incongruent easy | Incongruent  hard | Group | Condition | Condition x Group |
| Mid frontal | 0.88 (1.42) | 1.89 (1.69) | 1.82 (1.79) | 1.55 (1.40) | 2.59 (2.19) | 3.25 (2.09) | F_1,53=4.24; p=0.044 * | F_2.0,104.9=33.07; p=0.000 ** | F_2.0,104.9=3.09; p=0.050 |
| Lateral frontal | 0.44 (1.20) | 1.63 (1.58) | 1.70 (1.56) | 0.95 (1.24) | 1.94 (1.51) | 2.67 (1.64) | F_1,53=2.63; p=0.111 | F_1.8,96.4=60.59; p=0.000 ** | F_1.8,96.4=2.96; p=0.061 |
| Insula | 1.12 (1.78) | 2.81 (1.80) | 2.66 (2.06) | 1.30 (1.74) | 2.18 (2.04) | 2.80 (2.51) | F_1,53=0.04; p=0.839 | F_1.8,96.8=36.28; p=0.000 ** | F_1.8,96.8=2.85; p=0.067 |
| Parietal | 1.14 (1.25) | 2.57 (1.70) | 2.68 (1.76) | 1.91 (1.67) | 3.29 (2.17) | 4.01 (2.42) | F_1,53=3.70; p=0.060 | F_1.7,91.9=72.72; p=0.000 ** | F_1.7,91.9=2.36; p=0.107 |
| Occipital | 2.38 (1.88) | 3.68 (2.10) | 3.86 (2.37) | 2.82 (1.53) | 3.79 (2.03) | 4.60 (2.34) | F_1,53=0.66; p=0.421 | F_1.8,94.7=58.10; p=0.000 ** | F_1.8,94.7=2.08; p=0.135 |
| pDMN | 0.06 (1.62) | -0.79 (1.74) | -1.14 (2.05) | -1.66 (1.59) | -2.26 (1.81) | -2.62 (2.07) | F_1,53=11.14; p=0.002 ** | F_1.9,102.3=26.14; p=0.000 ** | F_1.9,102.3=0.43; p=0.642 |
| fDMN | -0.13 (1.24) | -0.60 (1.25) | -0.84 (1.39) | -1.44 (1.90) | -2.00 (2.40) | -2.12 (2.79) | F_1,53=6.61; p=0.013 * | F_2.0,103.6=10.51; p=0.000 ** | F_2.0,103.6=0.09; p=0.913 |
| Motor Cortex | 2.60 (1.39) | 3.29 (1.85) | 3.17 (1.95) | 3.09 (1.48) | 3.53 (1.54) | 3.61 (1.60) | F_1,53=0.85; p=0.361 | F_1.9,99.8=13.44; p=0.000 ** | F_1.9,99.8=0.61; p=0.533 |
| BG | 1.58 (1.50) | 2.59 (1.69) | 2.42 (1.81) | 1.25 (1.61) | 2.10 (2.24) | 2.57 (1.83) | F_1,53=0.24; p=0.628 | F_2.0,105.3=28.26; p=0.000 ** | F_2.0,105.3=2.26; p=0.110 |

** = p < 0.0056 (Bonferroni correction) * = p < 0.05

Table S3 BOLD latency for the 3 conditions or the SPM model with RT included as a parametric modulator.

|  | Control |  |  | LBD |  |  |  |  |  |
| --- | --- | --- | --- | --- | --- | --- | --- | --- | --- |
|  | N=23 |  |  | N=32 |  |  |  |  |  |
|  |  |  |  |  |  |  |  |  |  |
|  | Congruent | Incongruent easy | Incongruent  hard | Congruent | Incongruent easy | Incongruent  hard | Group | Condition | Condition x Group |
| Mid frontal | -0.05 (0.26) | -0.19 (0.28) | -0.17 (0.27) | -0.12 (0.26) | -0.13 (0.27) | -0.11 (0.28) | F_1,53_=0.06; p=0.815 | F_1.7,92.2_=2.92; p=0.066 | F_1.7,92.2_=2.49; p=0.096 |
| Lateral frontal | -0.02 (0.20) | -0.10 (0.17) | -0.09 (0.18) | 0.01 (0.18) | -0.01 (0.21) | 0.01 (0.21) | F_1,53_=3.04; p=0.087 | F_1.7,89.0_=1.51; p=0.228 | F_1.7,89.0_=0.94; p=0.380 |
| Insula | 0.02 (0.21) | -0.07 (0.21) | -0.02 (0.23) | 0.00 (0.21) | 0.08 (0.17) | 0.14 (0.22) | F_1,53_=4.47; p=0.039 * | F_1.9,102.5_=2.17; p=0.121 | F_1.9,102.5_=5.17; p=0.008 * |
| Parietal | -0.04 (0.25) | -0.15 (0.26) | -0.15 (0.31) | -0.03 (0.29) | 0.01 (0.34) | 0.13 (0.39) | F_1,53_=4.22; p=0.045 * | F_1.6,86.5_=1.50; p=0.229 | F_1.6,86.5_=6.38; p=0.005 ** |
| Occipital | -0.18 (0.29) | -0.19 (0.26) | -0.15 (0.29) | -0.10 (0.33) | 0.01 (0.31) | 0.16 (0.35) | F_1,53_=6.17; p=0.016 * | F_1.9,98.6_=12.49; p=0.000 ** | F_1.9,98.6_=8.32; p=0.001 ** |
| pDMN | 0.13 (0.16) | 0.12 (0.16) | 0.07 (0.20) | 0.07 (0.26) | 0.09 (0.25) | 0.07 (0.23) | F_1,53_=0.37; p=0.548 | F_1.9,101.7_=0.45; p=0.632 | F_1.9,101.7_=0.45; p=0.632 |
| fDMN | 0.07 (0.15) | 0.09 (0.16) | 0.10 (0.20) | -0.02 (0.23) | 0.03 (0.28) | 0.00 (0.26) | F_1,53_=3.24; p=0.077 | F_1.7,91.3_=0.65; p=0.504 | F_1.7,91.3_=0.19; p=0.794 |
| Motor Cortex | -0.38 (0.24) | -0.28 (0.26) | -0.26 (0.31) | -0.11 (0.33) | -0.02 (0.32) | 0.03 (0.32) | F_1,53_=13.18; p=0.001 ** | F_1.6,86.4_=11.00; p=0.000 ** | F_1.6,86.4_=0.09; p=0.881 |
| BG | -0.07 (0.20) | -0.14 (0.20) | -0.11 (0.15) | -0.01 (0.17) | 0.08 (0.18) | 0.07 (0.22) | F_1,53_=15.34; p=0.000 ** | F_2.0,103.4_=0.14; p=0.864 | F_2.0,103.4_=4.16; p=0.019 * |

** = p < 0.0056 (Bonferroni correction) * = p < 0.05

Table S4 Magnitude of the RT Parametric modulator in the SPM model with it included. Condition and group were not significant (all p >0.16).

|  | Control |  |  | LBD |  |  |  |
| --- | --- | --- | --- | --- | --- | --- | --- |
|  | N=23 |  |  | N=30 |  |  |  |
|  |  |  |  |  |  |  |  |
|  | Congruent | Incongruent easy | Incongruent  hard | Congruent | Incongruent easy | Incongruent  hard | Anova |
| Mid frontal | 0.50 (1.32) | 0.43 (1.20) | 0.83 (1.68) | 0.73 (2.48) | 0.56 (3.42) | 1.44 (5.54) | F_1,51_=10.80; p=0.002 ** |
| Lateral frontal | 0.52 (1.19) | 0.29 (1.26) | 0.68 (1.25) | 0.49 (1.89) | 0.59 (3.01) | 0.46 (3.62) | F_1,51_=7.84; p=0.007 * |
| Insula | 0.63 (1.50) | 0.97 (1.69) | 0.88 (1.29) | 0.28 (2.17) | -0.37 (7.48) | 1.57 (4.66) | F_1,51_=5.20; p=0.027 * |
| Parietal | 0.50 (1.25) | 0.32 (1.77) | 0.76 (1.25) | 0.67 (2.08) | 0.46 (2.81) | 0.03 (4.16) | F_1,51_=5.82; p=0.019 * |
| Occipital | 0.21 (1.43) | 0.55 (1.70) | 0.52 (1.08) | 0.50 (1.26) | 0.37 (3.04) | -0.41 (3.45) | F_1,51_=2.76; p=0.103 |
| pDMN | -0.41 (1.56) | -0.58 (2.02) | -0.59 (1.40) | -0.43 (1.75) | -1.42 (2.52) | -1.11 (3.36) | F_1,51_=22.05; p=0.000 ** |
| fDMN | -0.64 (1.46) | -0.37 (1.35) | -0.17 (1.28) | -0.72 (2.32) | -1.65 (4.57) | 0.81 (4.72) | F_1,51_=3.94; p=0.052 |
| Motor Cortex | 0.28 (1.03) | 0.28 (1.23) | 0.87 (1.30) | 0.30 (1.97) | 0.05 (2.53) | -0.60 (3.15) | F_1,51_=1.37; p=0.248 |
| BG | 0.25 (1.34) | 0.74 (1.89) | 0.57 (1.44) | 0.60 (1.34) | -0.65 (6.04) | -0.38 (2.79) | F_1,51_=0.54; p=0.465 |

** = p < 0.0056 (Bonferroni correction) * = p < 0.05
